# Reciprocal inhibition of the thigh muscles in humans: A study using transcutaneous spinal cord stimulation

**DOI:** 10.14814/phy2.16039

**Published:** 2024-05-13

**Authors:** Kento Nakagawa, Gaku Kakehata, Naotsugu Kaneko, Yohei Masugi, Rieko Osu, Shigeo Iso, Kazuyuki Kanosue, Kimitaka Nakazawa

**Affiliations:** ^1^ Faculty of Sport Sciences Waseda University Tokorozawa Saitama Japan; ^2^ Faculty of Human Sciences Waseda University Tokorozawa Saitama Japan; ^3^ Department of Sports and Health Management, Faculty of Business and Information Sciences Jobu University Isesaki Gunma Japan; ^4^ Graduate School of Arts and Sciences The University of Tokyo Meguro‐ku Tokyo Japan; ^5^ Japan Society for the Promotion of Science Chiyoda‐ku Tokyo Japan; ^6^ Department of Physical Therapy, School of Health Sciences Tokyo International University Kawagoe Saitama Japan; ^7^ Institute of Health and Sports Science and Medicine Juntendo University Inzai Chiba Japan

**Keywords:** hamstrings, posterior root muscle reflex, reciprocal inhibition, spinal cord stimulation, thigh

## Abstract

Evaluating reciprocal inhibition of the thigh muscles is important to investigate the neural circuits of locomotor behaviors. However, measurements of reciprocal inhibition of thigh muscles using spinal reflex, such as H‐reflex, have never been systematically established owing to methodological limitations. The present study aimed to clarify the existence of reciprocal inhibition in the thigh muscles using transcutaneous spinal cord stimulation (tSCS). Twenty able‐bodied male individuals were enrolled. We evoked spinal reflex from the biceps femoris muscle (BF) by tSCS on the lumber posterior root. We examined whether the tSCS‐evoked BF reflex was reciprocally inhibited by the following conditionings: (1) single‐pulse electrical stimulation on the femoral nerve innervating the rectus femoris muscle (RF) at various inter‐stimulus intervals in the resting condition; (2) voluntary contraction of the RF; and (3) vibration stimulus on the RF. The BF reflex was significantly inhibited when the conditioning electrical stimulation was delivered at 10 and 20 ms prior to tSCS, during voluntary contraction of the RF, and during vibration on the RF. These data suggested a piece of evidence of the existence of reciprocal inhibition from the RF to the BF muscle in humans and highlighted the utility of methods for evaluating reciprocal inhibition of the thigh muscles using tSCS.

## Graphical abstract

In this study, we used transcutaneous spinal cord stimulation (tSCS) to evoke spinal reflex from the biceps femoris (BF) muscle and demonstrated that the BF reflex was inhibited by several types of conditioning stimulations of sensory fibers of the antagonist rectus femoris muscle. Our data suggested a piece of evidence of the existence of reciprocal inhibition of the thigh muscles in humans.

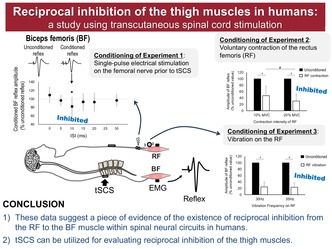

## 1 INTRODUCTION

The relationship in activation patterns between the agonist and antagonist muscles alters depending on the purpose of motor behavior or how the performed motor skill is sophisticated. The agonist and antagonist muscles show co‐activation when stiffness is required to stabilize movement or during the early phase of rehabilitation or motor adaptation (Biscarini et al., 2016; Franklin et al., 2008, 2012; Osu et al., 2002). However, in sophisticated smooth joint movements under stable environments, they are reciprocally activated in general (Burdet et al., 2001). That is, when the antagonist muscle is activated, the agonist muscle is inhibited at the spinal level, which is generally called as reciprocal inhibition (Pierrot‐Deseilligny & Burke, 2005). For example, reciprocal inhibition of the lower leg is modulated during motor behavior, such as walking or running, so that the agonistic motoneurons are suppressed during the activation phase of the antagonist muscle, which helps perform the desired movements smoothly (Petersen et al., 1999). Further, patients with neurological disorders, such as spinal cord injury, show reduced reciprocal inhibition compared to that observed in able‐bodied individuals (Okuma et al., 2002), which may be a factor of spasticity. The aforementioned issues suggest the functional importance of reciprocal inhibition for smooth motor behavior. Thus, reciprocal inhibition must be evaluated to understand the mechanisms underlying motor behavior and neurorehabilitation.

As a representative neural circuit of reciprocal inhibition, signal from the Ia‐sensory fibers of the antagonist muscle is sent to the spinal motoneuron innervating the agonist muscle via the Ia inhibitory interneuron. In human electro‐neurophysiological research, reciprocal inhibition is traditionally and widely evaluated using Hoffmann‐reflex (H‐reflex). H‐reflex can be evoked by electrical stimulation of the Ia‐sensory fibers at the peripheral nerve trunk. The H‐reflex amplitude reflects the monosynaptic spinal reflex excitability (Knikou, 2008). Typically, when the Ia‐sensory fibers of the antagonist muscle are activated beforehand, the H‐reflex amplitude is reduced. This reduction is considered as Ia reciprocal inhibition (Pierrot‐Deseilligny & Burke, 2005). As H‐reflex is usually utilized only in the plantarflexor or wrist flexor muscles owing to technical difficulty in stimulating Ia‐sensory fibers of the other muscles, reciprocal inhibition has been rarely investigated using H‐reflex except in the lower leg (Crone et al., 1987) and forearm (Day et al., 1984). Although reciprocal inhibition of the upper arm has been confirmed using the tendon reflex (Katz et al., 1991), less clear evidences supports the existence of reciprocal inhibition in the thigh muscles in humans (Pierrot‐Deseilligny & Burke, 2005). In an animal study, the reflex from the thigh muscles was suppressed during the activation phase of the antagonist muscles during locomotion (Gerasimenko et al., 2006). However, in humans, the reflexes of the thigh muscles are not reciprocally modulated during walking, whereas the reflexes of the lower leg show reciprocal modulation during the activation phase of antagonist muscles (Courtine et al., 2007). Indeed, the quadriceps and hamstring muscles appear to be activated at a similar timing (i.e., non‐reciprocal activation) in human walking (Capaday, 2002). Meanwhile, well‐trained sprinter runners show clear reciprocal activation of the thigh muscles during sprint running (Kakehata et al., 2021). Therefore, from previous studies showing the relationship between agonist and antagonist muscles during locomotion, it is unclear whether spinal neural circuits of reciprocal inhibition exist in the human thigh. A few previous studies have suggested the possibility of reciprocal inhibition of the thigh muscles during voluntary tonic muscle contraction in human (Bayoumi & Ashby, 1989; Hamm & Alexander, 2010). They showed that motor unit firing or electromyographic (EMG) signals from hamstring muscles were suppressed by femoral nerve stimulation of the antagonist quadriceps muscles. However, as the motor unit or EMG activity was recorded during voluntary contraction of the agonist hamstring muscles, the descending drive for neural inhibition of the agonist muscle was not eliminated in their study. As the modulation pattern of the excitability of the spinal reflex and corticospinal tract in the agonist and antagonist muscles during voluntary contraction is complicated (Saito et al., 2021a), it could not be concluded whether the suppression of the motor unit or EMG activity during voluntary contraction is caused by cortical or spinal neural circuits. To eliminate the supraspinal influence on reciprocal inhibition and examine the pure spinal contribution, it is necessary to investigate whether the excitability of the spinal motoneurons of the thigh muscles is suppressed by the external stimulation on the Ia‐sensory fibers of the antagonist muscle in the resting condition, not only during voluntary contraction. To the best of our knowledge, reciprocal inhibition from the quadriceps to hamstring muscles without voluntary contraction has never been investigated in detail using the established methods such as the spinal reflex which can be recorded even in the resting condition, as has been done in the lower leg and forearm, because of methodological limitations.

Recently, transcutaneous spinal cord stimulation (tSCS) has been used as a reliable tool for the evaluation of spinal reflex excitability from multiple muscles (Courtine et al., 2007; Minassian et al., 2007; Saito et al., 2019). To evoke spinal reflexes from the lower limb, such as the hamstring muscles, transcutaneous electrical stimulation is delivered over the lumber spinal cord to activate the Ia‐sensory fibers at the posterior root of the spinal cord. The evoked spinal reflex has been shown to have similar characteristics to H‐reflex. For instance, similar to the H‐reflex, the spinal reflex evoked by tSCS shows post‐activation depression both in thigh and lower leg muscles (Courtine et al., 2007). Further, the amplitude of spinal reflex evoked by tSCS is modulated by an sensory input, such as tendon vibration, passive movement, stretching of the muscle, or electrical stimulation (Courtine et al., 2007; Masugi et al., 2016, 2017), or during motor or cognitive tasks (Kaneko et al., 2019; Masugi et al., 2019; Nakagawa et al., 2018; Saito et al., 2021a, 2021b). Therefore, applying tSCS could clarify the reciprocal inhibition of the hamstring muscles, in which it is almost impossible to record H‐reflex.

In this work, we aimed to clarify the existence of reciprocal inhibition in the thigh muscles (especially, from the quadriceps to hamstring muscles) using the combination of tSCS and the three conditioning methods to activate the Ia‐sensory fibers of the antagonist muscle (rectus femoris): (1) single‐pulse electrical stimulation to Ia‐sensory fibers of the antagonist muscle (Crone et al., 1985; Day et al., 1984; Hirabayashi et al., 2019; Nakagawa et al., 2020) in the resting condition; (2) voluntary contraction of the antagonist muscle (Blazevich et al., 2012; Crone & Nielsen, 1989); and (3) muscle‐tendon vibration of the antagonist muscle (Cody & Plant, 1989). If the reciprocal inhibition between the thigh muscles exists, the reflex in the hamstring to the tSCS would be suppressed in all three conditions. Indeed, we provided evidence of reciprocal inhibition of the thigh muscles at the spinal level. The current method would become a new noninvasive tool for the study of motor control and for practical rehabilitation.

## 2 MATERIALS AND METHODS

### 2.1 Participants

Twenty able‐bodied male individuals (age, 21.3 ± 2.4 years; height, 175.2 ± 4.6 cm; weight, 67.0 ± 6.3 kg) were enrolled. Written informed consent was obtained from the participants. The research was approved by the Human Research Ethics Committee of Waseda University (approval number: 2019‐084). This study was conducted in accordance with the tenets of the Declaration of Helsinki. We did not recruit female individuals because of methodological limitations (i.e., the attached electrodes are on the skin surface of the trunk and groin).

### 2.2 EMG recording

Surface EMG signals were recorded at 2000 Hz using a Delsys Trigno EMG system (Delsys Inc., Natick, MA). The signals were band‐pass filtered between 10 and 850 Hz. EMG data were obtained from the right biceps femoris (BF) and rectus femoris (RF) muscles. We placed EMG sensors on the RF halfway along a line drawn from the anterior spina iliaca superior to the superior part of the patella, and on the BF halfway along a line drawn between the ischial tuberosity and the lateral epicondyle of the tibia (Hermens et al., 1999). Prior to attachment of the sensors, the involved area of the skin was shaved and treated with alcohol to reduce interelectrode impedance. EMG signals from the two muscles were checked after placing the electrodes. The signals were transferred to an A/D converter (PowerLab, AD Instruments, Sydney, Australia) and stored on a computer. The experimenters monitored the EMG signal online. When an EMG signal was observed during the resting condition (Experiment 1), the experimenters instructed the participants to relax and did not deliver stimulation until the EMG signals disappeared.

### 2.3 Spinal reflexes evoked by tSCS

The participants were in a supine position with their knee fully extended and both ankle joints were fixed by a strap to ankle foot orthoses at the neutral position to ensure the symmetrical position of the limbs. Spinal reflexes were recorded from the BF muscle with a constant‐current electrical stimulator with a single 200‐μs rectangular monophasic pulse (DS7AH, Digitimer Ltd., Welwyn Garden City, Hertfordshire, UK). A cathode (50 × 50 mm) was placed on the skin on the midline between spinous processes, and an anode (100 × 75 mm) was placed on the midline of the abdomen between the xiphoid process of the sternum and the umbilicus. The cathode was placed where single‐pulse stimulation produced the largest response of the BF muscle at the lower thoracic and upper lumbar vertebrae. The cathode was placed between L1 and L2 in all participants. The stimulation over L1 and L2 has been suggested to activate sensory fibers while the stimulation over the more caudal vertebrae such as L5 or S1 induces direct motor root activation (Roy et al., 2012). After determination of electrode placement, we used a double‐pulse stimulation paradigm (inter‐pulse interval: 50 ms) to confirm that the evoked responses originated in the sensory fibers, in accordance with previous studies (Courtine et al., 2007; Minassian et al., 2007; Nakagawa et al., 2018; Saito et al., 2021b). Using the double‐pulse stimuli, recruitment curves of the amplitude of the first and second responses evoked by tSCS were obtained to assess the stimulus intensity for each participant. The stimulus intensity was set so that the amplitude of the first response was on the ascending part of the recruitment curve (approximately 70% of the plateau amplitude) (Milosevic et al., 2019). The first reason for setting the stimulus intensity was that a large‐amplitude response, which reflects the excitability of a large number of spinal motoneurons, is suitable for capturing the degree of inhibition by the conditioning stimulus. Second, we avoided a higher tSCS intensity that induced the plateau amplitude because it often induces direct motor root activation (Roy et al., 2012). Furthermore, we set the stimulus intensity such that the post‐activation response in a double‐pulse paradigm includes minimal components originating from stimulation to the motor nerves. Thus, our stimulus intensity would be efficient for observing reciprocal inhibition. The amplitudes of the spinal reflexes at the stimulus intensity (138.8 ± 34.3 mA) during resting condition were compared between the first and second stimuli by paired *t*‐tests prior to Experiment 1 initiation. We conducted five double‐pulse tSCS trials at the determined stimulation intensity.

After determining the stimulus location and intensity, we conducted Experiment 1. Subsequently, Experiments 2 and 3 were conducted. Participants were allowed to rest ad libitum when they felt tired or sleepy.

### 2.4 Experiment 1: Effect of single‐pulse electrical stimulation of femoral nerve on the spinal reflex of the BF

**FIGURE 1:**
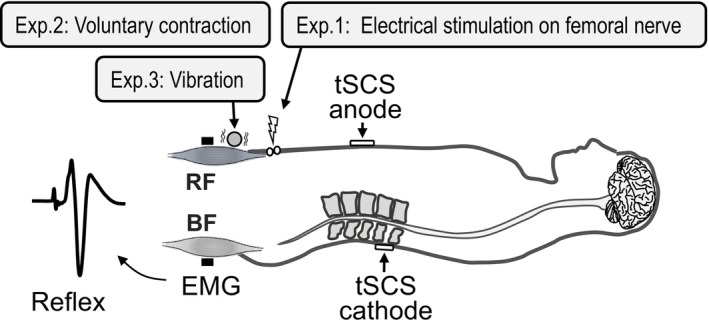
Experimental setup in Experiments 1–3. In Experiment 1, the conditioning electrical stimulation was delivered on the skin surface of the groin over the femoral nerve trunk (the stimulation electrodes are shown with white circles) to activate the Ia‐sensory fibers of the rectus femoris (RF) muscle. In Experiment 2, the participants conducted voluntary contraction of the RF muscle. In Experiment 3, muscle vibration on the RF muscle was delivered. During these conditionings to the RF muscle or unconditioned stimulation, transcutaneous spinal cord stimulation (tSCS) was commonly applied to evoke the spinal reflex from the biceps femoris (BF) muscle.

**FIGURE 2:**
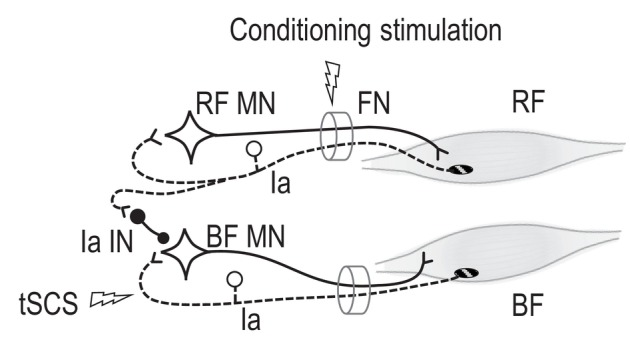
Sketch of the possible pathway of reciprocal inhibition from the rectus femoris (RF) to the biceps femoris motoneuron (BF MN) via reciprocal Ia interneuron (Ia IN) in Experiment 1. Conditioning stimulation was delivered on the RF Ia nerve at the femoral nerve trunk (FN). Spinal reflex of the BF was evoked by tSCS on the BF Ia fibers at the posterior nerve root.

Figures 1 and 2 illustrate the scheme of Experiment 1. To test reciprocal inhibition between the thigh muscles, electrical stimulation with a single pulse of 1 ms was applied on the femoral nerve to activate the Ia‐sensory fibers from the RF using an electrical stimulator (SEN‐7203, Nihon Kohden, Tokyo, Japan) as the conditioning stimulation. Using a pair of self‐made electrodes, which consisted of a bolt embedded in cork, we visually identified a site of the conditioning stimulation where the RF was contracted without contractions of the other muscles, such as the BF, adductor magnus, and others. The electrodes with 1‐cm diameter for the conditioning stimulation (inter‐electrodes interval: 2 cm) were located on the skin surface of the groin over the femoral nerve trunk. The motor threshold (MT) was defined as the minimum stimulation intensity to produce the RF muscle twitch that can be observed by visual inspection and palpation (Nakagawa et al., 2020). The intensities of the conditioning stimulation were set at 0.9 MT as the subthreshold of motor response but activating the Ia‐sensory fibers, and at 1.4 MT, at which the strong reciprocal inhibition was expected (Crone et al., 1985).

The inter‐stimulus intervals (ISIs) for the conditioning reflexes were set at 0, 5, 10, 15, 20, and 30 ms, which means that the test stimuli followed by the conditioning stimuli with these delays. A zero ISI indicates that the test and the conditioning stimuli were delivered at the same time. This ISI range was relatively larger than those associated with the traditional protocols examining reciprocal inhibition using soleus H‐reflex (e.g., −2 to 8 ms (Crone et al., 1987)) because of the following reasons. First, in this study, the difference in latencies of the conditioning stimuli on the groin and test stimuli on the lumber to reach the motoneuron of the BF should be larger than that of the above‐mentioned traditional protocol because of anatomical differences in the stimulus location. Second, the appropriate ISI (i.e., the time range, in which the reciprocal inhibition occurs) using tSCS had not been clarified. Thus, we needed an exploratory investigation to search the appropriate ISI in this paradigm. The ISIs were controlled using a pulse controller (SEN‐7203, Nihon Kohden, Tokyo, Japan) that could produce and precisely send pulses at a predetermined time.

The conditioned and unconditioned reflexes were obtained five times, respectively. The spinal reflexes evoked by the test stimulation without the conditioning stimulation (i.e., unconditioned reflex) were obtained prior to the conditioning‐test paradigms in each ISI. The ISI order was counterbalanced. For each participant, the values of the conditioned reflexes were normalized to the unconditioned value. Throughout Experiment 1, the participants were instructed to relax without any muscle contraction of the lower limbs. The experimenters carefully checked whether EMG signals of the lower limb muscles appeared or not.

For evaluation of the spinal reflex, the peak‐to‐peak amplitude within time windows from 5 to 45 ms after the tSCS was calculated (Masugi et al., 2017). The calculated amplitudes of the conditioned and unconditioned reflexes were averaged in each ISI. One‐sample *t*‐tests with Bonferroni correction were used to compare the normalized amplitudes of the conditioned reflex and 100% (i.e., unconditioned reflexes) for each ISI and conditioning intensity. Prior to the parametric statistical analysis, we confirmed whether the data set of conditioned reflexes was normally distributed by the Shapiro–Wilks test. As the data set at 0 and 2 ms ISI did not detect normality (*p* = 0.049 and *p* = 0.048, respectively), these were analyzed using non‐parametric Wilcoxon tests.

### 2.5 Experiment 2: Effect of voluntary contraction of the RF on the spinal reflex of the BF

In Experiment 2, participants conducted the isometric voluntary contraction of the RF (Figure 1). Voluntary contractions were conducted in two contraction intensities: 10% and 20% maximal voluntary contraction (MVC) of the RF. Prior to task initiation, we measured the magnitude of EMG activity of the RF during maximum isometric contraction of the RF (i.e., MVC) for approximately 3 s two times. Participants were instructed to perform isometric hip flexion in the contraction task and MVC. Based on the higher EMG signal amplitude of the MVC trials, we set the target line at 10% and 20% MVC. The participants were provided verbal feedback of the degree of muscle contraction of the RF online. tSCS was delivered when the RF EMG signal amplitude kept stable around the target level. Once tSCS was delivered, the participants stopped muscle contraction and relaxed. Prior to conducting the contraction tasks, the unconditioned spinal reflexes were obtained at resting condition five times. Then, the conditioned spinal reflexes were obtained during the task five times. The order of the tasks (10% and 20% MVC) was counterbalanced.

The calculated peak‐to‐peak amplitudes both of conditioned and unconditioned reflexes were averaged in each task. Using the Shapiro–Wilks tests, we confirmed the normal distribution of the data set of amplitude of the conditioned reflexes and employed one‐sample *t*‐tests with Bonferroni correction to compare the reflex amplitude normalized to its unconditioned value during the task with the 100% for each contraction intensity. Further, the conditioned amplitudes normalized to the unconditioned value were compared between the 10% and 20% MVC tasks. The root mean square of the EMG signals immediately before tSCS (50‐ms window) were calculated as background muscle activity (BGA) for the contraction task. The BGA values of the BF and RF during the tasks and resting condition were compared using non‐parametric analysis because the Shapiro–Wilks tests showed a non‐normal distribution for the BGAs of the RF at 10% (*p* = 0.012), BF at 10% (*p* < 0.001) and 20% (*p* < 0.001), and resting condition (*p* < 0.001). For analyzing these non‐normality data, non‐parametric Wilcoxon tests were performed when the Friedman tests reached a significance.

### 2.6 Experiment 3: Effect of muscle vibration over the RF on the spinal reflex of the BF

As the mechanical vibration to a muscle is also representative stimulation on the Ia‐sensory fibers, we investigated whether the reciprocal inhibition from the RF to BF appears by vibration to the RF muscle (Figure 1). Regarding the vibration stimuli, we used a vibrator, which can change vibration frequency (B09Q7XM1K4, Topersun). Vibration frequency was set at 30 and 55 Hz to investigate whether the reciprocal effect is dependent on vibration frequency. These used frequencies were the minimum and maximum frequencies of this vibrator. A vibrator was placed on the skin over the RF (proximal side) using stands with a movable arm (C‐stand, Avenger, Cassola, Italy) to avoid changing the load to the skin. The load pressure was not measured but the vibrator was fixed to the extent that it just touched the skin. Prior to the vibration, the unconditioned spinal reflexes were obtained without vibration five times. At the measurement without vibration, the vibrator kept attached on the skin with the vibration turning off. Then, the vibrator turned on and the spinal reflexes were obtained during the vibration five times (i.e., conditioned reflex). The order of the vibration frequency (30 and 55 Hz) was counterbalanced.

The calculated amplitudes of the conditioned and unconditioned reflexes were averaged in each vibration frequency. Using the Shapiro–Wilks test, we confirmed the normal distribution of the conditioned reflex‐amplitude dataset and employed one‐sample t‐tests with Bonferroni correction to compare the conditioned reflex amplitude normalized to its unconditioned value during the task with the 100% for each contraction intensity. Shapiro–Wilks tests did not find the normality of the BGAs both of the RF (30 Hz, *p* = 0.002; 55 Hz, *p* < 0.001) and RF muscles (30 Hz, *p* < 0.001; 55 Hz, *p* < 0.001; and without vibration; *p* < 0.001). Thus, the BGAs of the RF and BF muscles were analyzed using non‐parametric Wilcoxon tests when the Friedman tests detected a significance. The level of significance was set at p < 0.05.

## 3 RESULTS

**FIGURE 3:**
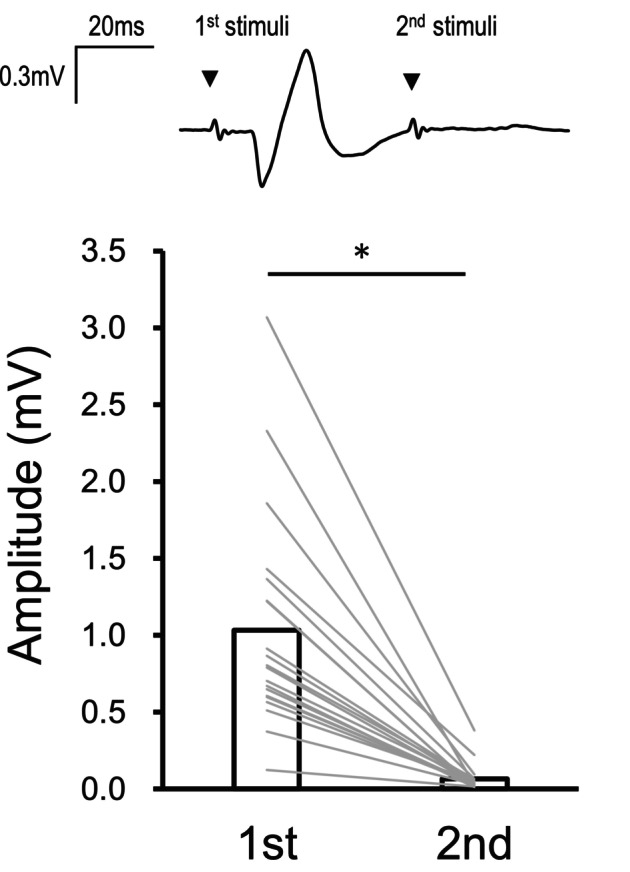
Amplitude of responses to the first and second stimulation in double‐pulse paradigm (50 ms interval). Upper waveform represents the typical recordings of elicited responses from the biceps femoris (BF) muscle evoked by double‐pulse stimulation. These data were obtained from a single participant. The timings of the first and second stimulus are shown with black triangles. Note that the response after the second stimulus almost disappears. The graph shows the quantified group data of amplitudes of the first and second responses. White bars indicate the mean value. Gray lines represent the individual datapoints. An asterisk indicates the significant difference in amplitude of the response between the first and second responses.

Using double‐pulse stimuli at the determined stimulus intensity, we confirmed that the amplitude of the second responses induced by double‐pulse tSCS in a resting state were dramatically and significantly suppressed from the first responses (6.3 ± 3.9% of the first response, *t* [19] = 6.67, *p* < 0.001) (Figure 3) owing to post‐activation depression (Pierrot‐Deseilligny & Burke, 2005), indicating that tSCS activated Ia‐sensory fibers and elicited a monosynaptic response (i.e., spinal reflex).

### 3.1 Experiment 1

**FIGURE 4:**
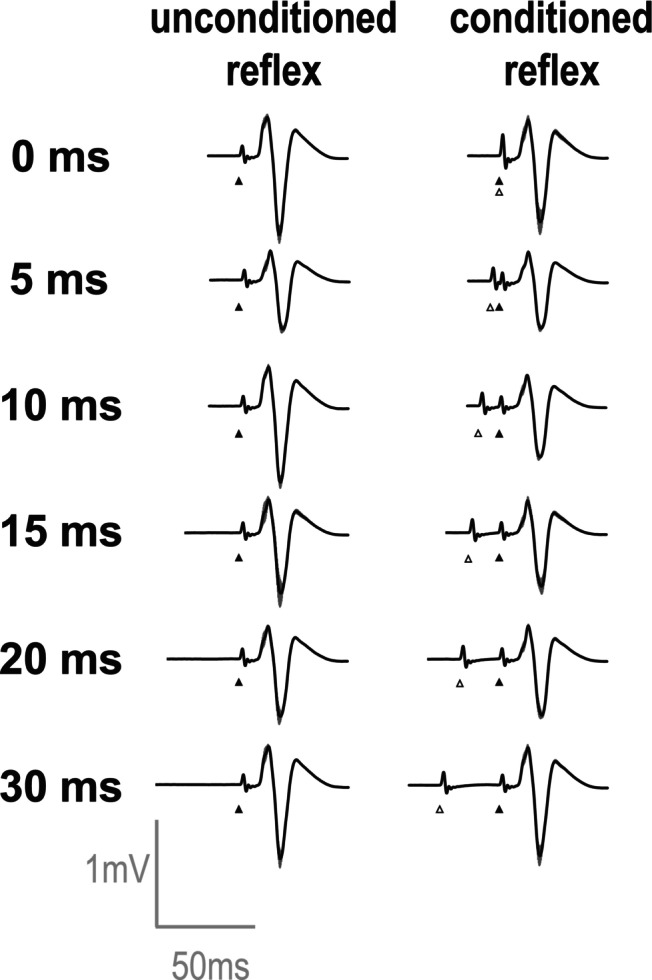
Representative waveforms of the spinal reflexes from the biceps femoris (BF) muscle in Experiment 1 (1.4 × motor threshold [MT]) from a single participant. In each inter‐stimulus interval (ISI), unconditioned reflex (i.e., without conditioning stimulation) was shown in the left side and conditioned reflex (i.e., conditioning electrical stimulation on femoral nerve) was shown in the right side. Five waveforms were overlaid with different color. A black triangle indicates the timing of transcutaneous spinal cord stimulation (tSCS) while a white triangle represents the timing of the conditioning stimulation on the femoral nerve. In this participant, the spinal reflex was prominently suppressed at the 10 ms ISI.

**FIGURE 5:**
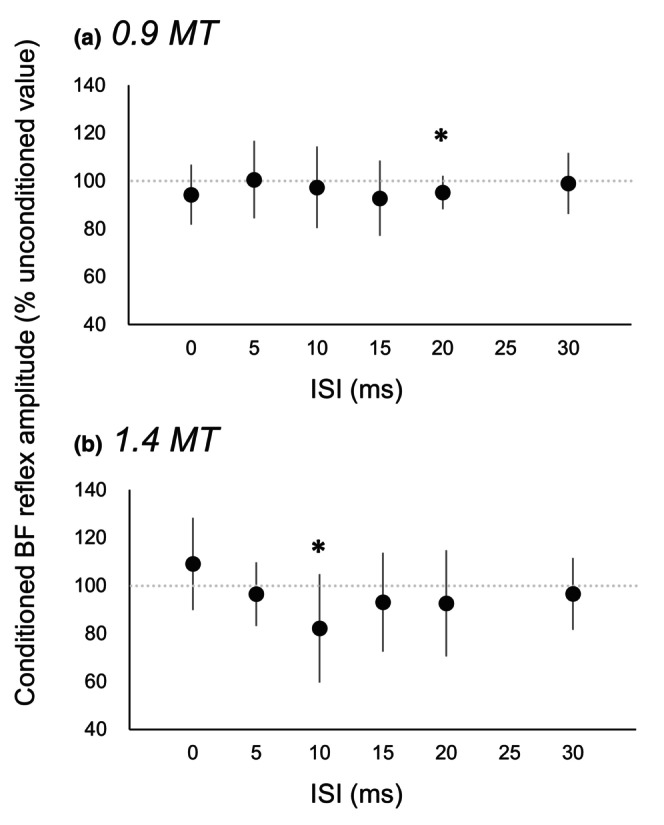
Changes in the conditioned biceps femoris (BF) reflex amplitude normalized to unconditioned value depending on inter‐stimulus interval (ISI) in Experiment 1. (a) Conditioning stimulation intensity was 0.9 (a) and 1.4 (b) × motor threshold (MT) of the rectus femoris (RF) muscle. Asterisks indicate the significant difference between the conditioned and unconditioned value. Error bars represent standard deviation (SD).

Figure 4 shows the averaged waveforms of the conditioned and unconditioned reflexes in the BF for 1.4 MT of conditioning stimulus in a representative participant. The reflex amplitude at 10‐ms ISI appears to become smaller compared to the unconditioned reflex. Figure 5 expresses the relative values of amplitudes of the conditioned reflexes to the unconditioned reflexes in the BF for each ISI (A, 0.9 MT; B, 1.4 MT). For 0.9 MT, the amplitudes of conditioned reflexes in the BF were significantly lower than those of unconditioned reflexes only when the ISI was 20 ms (95.2 ± 7.1%; *t* [19] = 3.00, *p* = 0.04). In the other ISIs (0, 2, 5, 10, 15, and 30 ms), there were no significant differences in amplitudes between the conditioned and unconditioned reflexes. For 1.4 MT, the amplitudes of conditioned reflexes in the BF were significantly lower than those of unconditioned reflexes only when the ISI was 10 ms (81.3 ± 22.8%; *t* [19] = 3.68, *p* = 0.01). In the other ISIs (0, 2, 5, 15, 20, and 30 ms), there were no significant differences in amplitudes between the conditioned and unconditioned reflexes.

### 3.2 Experiment 2

**FIGURE 6:**
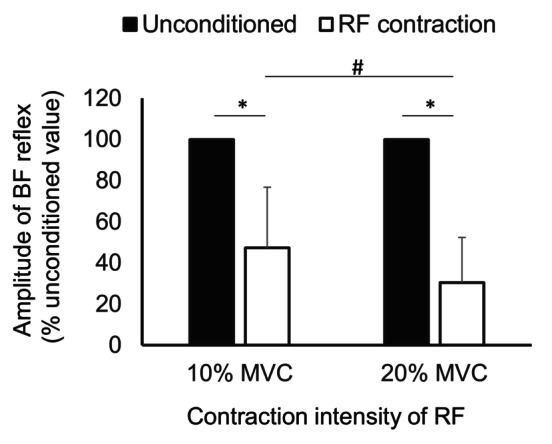
Changes in the biceps femoris (BF) reflex amplitude during voluntary contraction of the rectus femoris (RF) in Experiment 2. Reflex amplitude is expressed as a percentage of the unconditioned reflex amplitude recorded prior to the voluntary contraction. Asterisks indicate the significant difference between the conditioned (voluntary contraction of the RF muscle) and unconditioned (rest) value. Hash represents the significant difference in the conditioned values between the 10% and 20% maximal voluntary contraction tasks. Error bars represent standard deviation (SD).

Figure 6 represents the relative amplitude of spinal reflexes from the BF during contracting the RF muscle and resting conditioning (i.e., unconditioned reflex = 100%). The conditioned reflex amplitude was smaller than unconditioned value both in the 10% (*t* [19] = 7.41, *p* < 0.001) and 20% MVC tasks (*t* [19] = 13.28, *p* < 0.001). Furthermore, the amplitude was significantly smaller in the 20% than in the 10% MVC task (*t* [19] = 2.41, *p* = 0.026).

For the BGA of the RF, the mean values normalized to that of the MVC (±SD) were 11.5 ± 3.1%, 18.3 ± 2.8%, and 2.0 ± 1.4% for the 10% MVC task, 20% MVC task, and resting condition, respectively. Thus, participants could control the contraction intensity approximately as instructed. Indeed, the Friedman test revealed a significant difference across the three conditions (*p* < 0.001). Subsequently, posthoc Wilcoxon tests found that there were significant differences in the BGA between 10% MVC and rest (*p* < 0.001), 20% MVC and rest (*p* < 0.001), and 10% MVC and 20% MVC (*p* < 0.001). For the BGA of the BF, the mean values of %MVC (± SD) were 2.9 ± 3.1%, 3.75 ± 6.12%, and 2.01 ± 1.38% for the 10% MVC task, 20% MVC task, and resting condition, respectively. The Friedman test revealed significant differences across the tasks (*p* = 0.004). Wilcoxon tests detected a significant difference in the BGA of the BF between the 10% MVC task and the resting condition (*p* = 0.045), and between the 20% MVC task and the resting condition (*p* < 0.001). There was no difference between the 10% and 20% MVC tasks (*p* = 0.08).

### 3.3 Experiment 3

**FIGURE 7:**
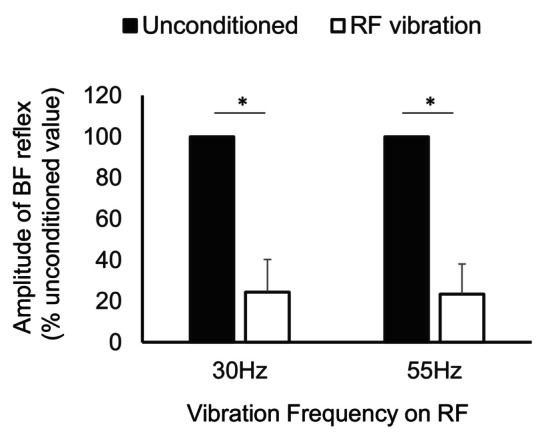
Changes in the biceps femoris (BF) reflex amplitude during muscle vibration on the rectus femoris (RF) in Experiment 3. Reflex amplitude is expressed as a percentage of the unconditioned reflex amplitude recorded prior to the vibration. Asterisks indicate the significant difference between the conditioned (vibration on the RF muscle) and unconditioned value (no vibration). Error bars represent standard deviations (SDs).

Figure 7 represents the relative amplitude of spinal reflexes of the BF during muscle vibration on the RF muscle and without vibration (i.e., unconditioned reflex = 100%). During the muscle vibration over the RF, the amplitude of spinal reflexes from the BF muscle significantly decreased from the unconditioned value (i.e., reflex amplitude without vibration) both in 30 Hz (*t* [19] = 20.13, *p* < 0.001) and 55 Hz conditions (*t* [19] = 21.88, *p* < 0.001). There was no significant difference between the 30 and 55 Hz (*t* [19] = 2.41, *p* = 0.052) conditions.

Regarding the BGA of the RF, the Friedman test detected a statistical significance (*p* < 0.001). Subsequently, the Wilcoxon tests revealed significantly higher BGAs of the RF during the RF vibration compared to the unconditioned values for the 30 Hz (*p* < 0.001) and 55 Hz frequencies (*p* < 0.001). There was no significant difference in the BGAs between the 30 and 55 Hz (*p* = 0.09) conditions. Concerning the BGAs of the BF, the Friedman test did not detect a significance (*p* = 0.68).

## 4 DISCUSSION

In this study, we aimed to clarify the existence of reciprocal inhibition in the thigh muscles within spinal neural circuits using tSCS. Our findings showed that the BF spinal reflex was suppressed by prior electrical stimulation to the femoral nerve innervating the RF muscle, voluntary contraction of the RF muscle, and vibration stimuli to the RF muscle. These results suggest the reciprocal inhibition between the thigh muscles in human can be evaluated by using tSCS in a similar way to the traditional methods using H‐reflex on lower leg muscles (e.g., soleus). As tSCS can evoke spinal reflex from various lower‐limb muscles, reciprocal inhibition may be measured from muscles other than soleus that has been used in previous studies. This can extend the research field of spinal reciprocal inhibition. Further, one of the strengths of tSCS is that spinal reflex excitability can be measured irrespective of whether the muscle is voluntarily contracted or resting. Therefore, tSCS can distinguish supraspinal from within spinal effects on reciprocal inhibition. As supraspinal neural circuits would not be mediated for Experiments 1 and 3, the observed reciprocal inhibition between the thigh muscles should be generated within spinal neural circuits.

In Experiment 1, a significant inhibition of the BF reflex was observed when the ISI was 10 ms for 1.4 MT and 20 ms for 0.9 MT. For 1.4 MT, the mean value of the conditioned BF reflex was smaller than that of the unconditioned BF reflex at 15 and 20 ms ISIs, although these were not significant. Thus, the suppression of the BF spinal reflex appears to occur when the ISI was approximately 10–20 ms. Therefore, the question that raises is whether these ISIs are reasonable for producing reciprocal inhibition. To answer this question, it is necessary to estimate the timing difference in the arrival of pulses derived from conditioning and test stimuli to the BF motor neurons. We estimated the followings:

1. The conduction time from femoral nerve stimulation to the BF motoneuron via Ia reciprocal inhibitory interneuron using the following parameters.
2. The conduction time from the posterior root nerve at the vertebral foramina to the RF muscle (i.e., the latency of the RF spinal reflex induced by tSCS): 9 ms.
  The aforementioned value was calculated as follows: we detected the latency from the tSCS and onset of the RF response by visual inspection in the resting condition in each participant and regarded this value as the conduction time from the posterior root nerve to the RF muscle (mean ± SD, 9.2 ± 1.3 ms).
3. The conduction time from the femoral nerve at the groin to the RF muscle (i.e., the latency of the RF H‐reflex) induced by femoral nerve stimulation: 17.5 ms (reported by Garland et al. (1994)).
4. The conduction time from the posterior root nerve at the vertebral foramina to the BF motoneuron through the monosynaptic reflex pathway estimated by Taylor and Martin (2009) and used in previous works (Bunday & Perez, 2012; Dixon et al., 2016; Taylor & Martin, 2009): 1.5 ms.
5. The conduction time from posterior root nerve at the vertebral foramina to the BF motoneuron through reciprocal inhibitory interneuron: 2 ms ([4] + 0.5 ms, where 0.5 ms was the estimated time of synapse transmission of the interneuron (Taylor & Martin, 2009)).

Thus, we calculated as follows: [1] = [3] – [2] + [5] = 10.5 ms.

Based on these calculated parameters, in case of 10 ms ISI in Experiment 1, the input of reciprocal inhibition induced by conditioning stimulation on the femoral nerve arrived at the BF motoneuron at 1 ms prior to the input of impulse by tSCS. This means that the amplitude of the BF spinal reflex induced by tSCS would reflect the excitability of the BF motoneuron at 1 ms after initiating reciprocal inhibition on the BF motoneuron. This timing would be reasonable to explain the influence of the Ia reciprocal inhibitory interneuron as the following reasons. In previous works using traditional methods to measure the reciprocal inhibition using soleus H‐reflex, the reciprocal inhibition was observed even when the timing difference arriving at soleus motoneuron was 1–2 ms and lasted for approximately 3–4 ms (Crone et al., 1987; Pierrot‐Deseilligny & Burke, 2005). Thus, the observed significant inhibition of the BF reflex at 10 ms ISI in 1.4 MT is probably attributed to Ia reciprocal inhibition from the RF Ia‐sensory fibers. The absence of significant inhibition at 10 ms ISI in 0.9 MT might be attributed to insufficient input from the Ia‐sensory fibers to produce reciprocal inhibition. In contrast, the significant inhibition of the BF reflex was also observed at 20 ms ISI in 0.9 MT. The timing difference arriving the BF motoneuron would be 11 ms in case of 20 ms ISI. This seems to be too late for the effective duration of Ia reciprocal inhibition considering the previously reported data. The observed inhibition at 20 ms ISI could be attributed to mechanisms through oligo or polysynaptic pathway other than Ia reciprocal inhibition, such as D1 inhibition (El‐Tohamy & Sedgwick, 1983) or the inhibition of interneuron originated from Ib projection of the antagonist muscle (Pierrot‐Deseilligny et al., 1981). Indeed, other than Ia reciprocal inhibition, the duration of these inhibitory circuits has been reported as five to several 10 ms (El‐Tohamy & Sedgwick, 1983; Pierrot‐Deseilligny et al., 1981). Although 0.9 and 1.4 MT conditions showed a trend of inhibition, there was no significant inhibition at 15 ms ISI, which may be attributed to the fact that the effect of these inhibitions might not have been sufficiently induced at that timing. Especially, the timing of 15 ms ISI might have been located between the Ia reciprocal inhibition and the inhibitions of the other mechanisms. Overall, the 10 and 20 ms ISIs where the reciprocal inhibition appeared in this study could be reasonable and correspond to previous studies using traditional methods targeting lower leg or forearm muscles. Meanwhile, it is hard to explain why there was no significant inhibition at 20 ms ISI in 1.4 MT while the stimulation of 0.9 MT intensity inhibited. As the stimulation of 1.4 MT would activate more various neural circuits than that of 0.9 MT, the inhibitory effects may be masked by some neural circuits enhancing the excitability of the BF motoneuron. We need more researches to investigate the detailed mechanisms of the stimulus‐intensity dependent difference. Furthermore, as we needed exploratory tests, the present study investigated the reciprocal inhibition with ISIs of 5 ms increments from 5 to 20 ms ISI, which would be a little bit rough. In the future, to investigate the more detailed time series of change in pattern of reciprocal inhibition of the thigh muscles, it would be necessary to measure the reciprocal inhibition with smaller steps of ISIs, such as 1 ms of ISI increments in the range between 5 and 25 ms ISIs. As a limitation of the above estimation of the timing difference, we only estimated the conduction time from the latency of response onset and previously reported conduction time but did not use conduction distance and velocity. Furthermore, the same conduction time was used across participants of different heights. Therefore, our calculation of the estimated conduction time is likely to be rough and only for reference purposes.

In Experiment 2, the BF spinal reflex was significantly inhibited during voluntary contraction of the RF both of 10% and 20% MVC. Additionally, the magnitude of inhibition was higher in 20% than in 10% MVC. These results are in agreement with those of previous studies using H‐reflex (Crone et al., 1987; Nielsen & Kagamihara, 1993) and our study that investigated the changes in spinal reflex induced by tSCS associated with voluntary contraction at various body parts (Saito et al., 2021a). Our results suggest the usefulness of tSCS for examination of reciprocal inhibition of the thigh muscles during voluntary contraction or motor tasks. Stronger inhibition (i.e., >50%) of its rest value, induced by RF contraction compared to the conditioning electrical stimulation in Experiment 1 (81% of unconditioned value at the most) would be affected by various factors in addition to Ia reciprocal inhibition from the antagonist muscle. For example, descending drive is supposed to have a main contribution to facilitate the Ia interneuron via direct connection from corticospinal pathway or propriospinally mediated inhibition (Crone et al., 1987; Pierrot‐Deseilligny & Burke, 2005). Branched descending drive to the BF muscle and accompanying the recurrent inhibition of the BF would be also possible factors for inhibition of the BF spinal reflex during the RF contraction, as there were small EMG activities in the BF during the RF contraction. Therefore, as estimated, the experimental paradigm with descending drive would inevitably contain the supraspinal effects on the reciprocal inhibition. Specifically, the supraspinal effects accompanied with descending drive appears to strengthen the reciprocal inhibition.

In Experiment 3, the BF spinal reflex during vibration on the RF muscle was also prominently inhibited. A behavioral study showed that prolonged vibration on knee extensor neither changed the maximum voluntary isometric knee flexion torque nor activation the knee flexors during squat exercise (Ema et al., 2017). Thus, it remains unclear whether vibration stimuli can induce reciprocal inhibition in the thigh muscles, but the present study clearly suggested vibration‐induced reciprocal inhibition between the thigh muscles. A previous work stated that vibration on plantarflexors inhibited the excitability of tSCS‐evoked spinal reflex of the dorsiflexor (Minassian et al., 2007). However, to our knowledge, no study has demonstrated the reciprocal inhibition of thigh muscles induced by vibration until the present study showed. Therefore, this experiment using vibration also suggests the utility of evaluation of reciprocal inhibition within thigh muscles using tSCS.

Muscle vibration is supposed to stimulate both Ia and Ib sensory fibers (Ema et al., 2017). As Ib inhibition has been suggested to work not only on the agonist and synergistic muscles but also on the antagonist muscle (Cardinale & Bosco, 2003; Yanagawa et al., 1991), the effect of Ib inhibition would contribute to the inhibition of the BF spinal reflex induced by the RF vibration in addition to Ia reciprocal inhibition in the present study. Furthermore, though the vibrator used was set on the RF surface and the intensity was not strong because of no compression and there was no difference in background EMG of the BF with and without vibration on the RF, vibration might reach to the BF muscle, which might result in Ib inhibition from the BF Golgi tendon organs to the BF muscle in some extent. The strong inhibition of the BF reflex induced by the RF vibration (approximately 15% of its non‐vibrated value) would be affected by these various factors.

Meanwhile, there was no difference in inhibition of the BF reflex nor background EMG of the RF muscle between 30 and 55 Hz frequencies, which were the minimal and maximal frequencies of utilized equipment. Thus, the reciprocal inhibition of the BF and tendon vibration reflexes of the RF induced by muscle vibration may not depend on the vibration frequency at least within the range from 30 to 55 Hz.

The present study suggested that the reciprocal inhibition from the RF to BF muscle was likely observed when utilizing the tSCS and the three types of conditionings: single‐pulse electrical stimulation, voluntary contraction, and vibration stimulation. To date, reciprocal inhibition of thigh muscles in resting condition in humans has not been well investigated and clearly demonstrated whether it can be observed or not in resting condition, while an animal study suggests the existence of Ia inhibition from knee extensor to flexor (Eccles & Lundberg, 1958). Overall, these data indicate the existence of reciprocal inhibition of thigh muscles in humans within spinal neural circuits and suggest that the reciprocal inhibition of thigh muscles using tSCS appears to have similar characteristics to the reciprocal inhibition of lower leg or forearm using traditional H‐reflex methods. As tSCS is a noninvasive method to evoke spinal reflex and can be applied to several patients, such as those spinal cord injury (Hofstoetter et al., 2019), the methods inducing reciprocal inhibition of the thigh muscles using tSCS could be useful for evaluating function of reciprocal inhibition in these patients as well. Especially, as the present study suggested that reciprocal inhibition of the thigh muscles can be evaluated even in resting condition, our proposed methods should be applicable to patients who have paralysis or injury of thigh muscles. As the previous studies that observed the reciprocal inhibition of the thigh muscles required participants to conduct voluntary contraction (Bayoumi & Ashby, 1989; Hamm & Alexander, 2010), it is clinically significant to demonstrate that the reciprocal inhibition of the thigh muscles can be measured irrespective whether the muscles are contracted or resting for patients with physical disabilities. Furthermore, the proposed methods can be applied to measuring the neural function of able‐bodied athletes. For example, well‐trained sprinters show smooth switching of the agonist–antagonist muscle activities in thigh, which results in decreasing the co‐contraction of the agonist and antagonist muscles (Kakehata et al., 2021). Reciprocal inhibition of the thigh muscles can be considered to contribute to this specific activation patterns of the thigh muscles in sprinters. In the future, we should evaluate the neural circuits of reciprocal inhibition of thigh muscles using tSCS in patients or athletes, which might lead to functional improvement of their locomotor ability or sports performance.

There are some limitations in the present study. First, owing to methodological limitations, only male participants in the present study were included. However, it is unclear whether different results can be found in males and females. Second, from the data of the double‐pulse tSCS paradigm, we confirmed that the evoked potential elicited by tSCS mainly originated from the Ia‐sensory fibers. However, we cannot eliminate the possibility that the components of direct motor root activation were present, as the second response in the double‐pulse tSCS was not completely suppressed (i.e., the degree of suppression was 93.7%). Thus, the components of direct motor root activation might have been slightly contained in the tSCS‐evoked potentials. Meanwhile, the stimulation site in the current study (i.e., L1‐L2) has been suggested to mainly activate sensory fibers, unlike the more caudal vertebrae (e.g., L5‐S1) in which stimulation mainly activates motor fibers (Roy et al., 2012). Therefore, our stimulation parameters were appropriate for efficiently evoking spinal reflexes.

### 4.1 Conclusion

Our study demonstrated that the spinal reflex of hamstring muscle was inhibited by single‐pulse electrical stimulation on femoral nerve of the antagonist muscle at the specific inter‐stimulation intervals in the resting state, voluntary contraction, and vibration of the antagonist muscle. These data suggest a piece of evidence of the existence of reciprocal inhibition of the thigh muscles within spinal neural circuits in humans and utility of the methods for evaluating reciprocal inhibition of the thigh muscles using tSCS.

## AUTHOR CONTRIBUTIONS

K.N., G.K., and N.K. conceived and designed research; K.N. and G.K. performed experiment; K.N. and G.K. analyzed data; K.N., G.K., N.K., and Y.M. interpreted results of experiments; K.N. and G.K. prepared figures; K.N. and G.K. drafted manuscripts; K.N., G.K., N.K., Y.M., R.O., S.I., K.K., and K.N. edited and revised manuscript; K.N., G.K., N.K., Y.M., R.O., S.I., K.K., and K.N. approved final version of manuscript.

## FUNDING INFORMATION

This study was supported by Grant‐in‐Aid for Scientific Research (B) (JSPS KAKENHI Grant Number 22H03498) to K. Nakagawa and Grant‐in‐Aid for JSPS Fellows (JSPS KAKENHI Grant Number 22KJ0713) to G. Kakehata.

## CONFLICT OF INTEREST STATEMENT

None declared.

## ETHICS STATEMENT

The research was approved by the Human Research Ethics Committee of Waseda University (approval number: 2019‐084). This study was conducted in accordance with the tenets of the Declaration of Helsinki.

## Data Availability

Data are available from the corresponding author upon reasonable request.
